# Closed Complete Annotated Genome Sequences of Five Haemophilus influenzae Biogroup *aegyptius* Strains

**DOI:** 10.1128/MRA.01198-19

**Published:** 2019-11-07

**Authors:** Zachary N. Phillips, Greg Tram, Michael P. Jennings, John M. Atack

**Affiliations:** a Institute for Glycomics, Griffith University, Gold Coast, Queensland, Australia; Queens College

## Abstract

Haemophilus influenzae biogroup *aegyptius* is a cause of conjunctivitis in children. Biogroup *aegyptius* strains also caused fatal outbreaks of invasive disease, known as Brazilian purpuric fever (BPF), in the 1980s. BPF is fatal if untreated. Here, we report the complete genome sequences of five strains of Haemophilus influenzae biogroup *aegyptius*.

## ANNOUNCEMENT

Haemophilus influenzae biogroup *aegyptius* (1) is a bacterial pathogen responsible for purulent conjunctivitis in young children. It was also the cause of the acute and deadly invasive infection Brazilian purpuric fever (BPF) in the 1980s (2). It was hypothesized that acquisition and/or expression of particular virulence factors led to the emergence of BPF strains (3, 4), but the exact mechanism(s) behind the transition from causing conjunctivitis to causing severe invasive disease was never elucidated. However, a number of Haemophilus influenzae biogroup *aegyptius*-specific virulence factors have been identified and studied (5), such as lipooligosaccharide structure (1), extracellular proteins (6), and outer membrane proteins/adhesins (4, 7). Nevertheless, no factors were conclusively shown to be completely necessary for virulence (1), in part due to a lack of high-quality genome sequences. Prior to this study, only four closed annotated genome sequences for Haemophilus influenzae biogroup *aegyptius/*Haemophilus aegyptius (NCBI taxonomy identification [ID] 725 and ID 197575) were available in the public domain (strains F3031 [GenBank accession number FQ670178], F3047 [FQ670204], NCTC8134 [LR134395], and NCTC8502 [LS483429]). Another 10 strains have been deposited as whole-genome shotgun (WGS) contigs. Herein, we report the closed annotated whole-genome sequences of five biogroup *aegyptius* strains, BPF isolates F1946, F3028, and F3037 and conjunctivitis (non-BPF) isolates F3043 and F3052. All five strains were originally isolated in Brazil in the 1980s (3).

Bacterial strains were grown on brain heart infusion (BHI) agar supplemented with NAD^+^ (2 μg/ml) and hemin (1% [vol/vol]). Genomic DNA was prepared using the GenElute kit (Sigma-Aldrich) and sequenced at the Australian Genome Research Facility (AGRF) using the PacBio Sequel platform with P6-C4 chemistry and a library size of 10 kb, with barcoded libraries and a 10-hour read time using a single Sequel single-molecule real-time (SMRT) cell. Sequence reads were filtered and genomes were *de novo* assembled and polished using the hierarchical genome assembly process (HGAP) version 4 (8) using default settings, except that the genome size was set at 1,900,000 bp. The assembly quality was assessed using BUSCO analysis (9). Whole closed genome sequences for each strain were submitted to NCBI for annotation using the Prokaryotic Genome Annotation Pipeline (PGAP). The complete annotated closed genomes have been deposited in GenBank.

Information for each strain/genome is summarized in Table 1. All five strains contain a number of genes associated with Haemophilus influenzae biogroup *aegyptius* (4), including high-molecular-weight (HMW) adhesins containing an octanucleotide 5′-GCATCATC_[n]_-3′ repeat in their promoter region (4) and a number of biogroup *aegyptius*-specific trimeric autotransporter proteins (4). Four of the five strains (F1946, F3028, F3043, and F3037) contain an ∼32.5-kb plasmid (GenBank accession number AF447808) associated with Haemophilus influenzae biogroup *aegyptius* strains (10). Interestingly, our sequence of strain F1946 is ∼28 kb larger than the WGS sequence deposited recently (11). The extra sequences, able to be resolved here due to long-read sequencing, consist of a number of duplicated regions, such as rRNA gene clusters (∼5.5 kb each), the *licABC* operon involved in lipooligosaccharide (LOS) biosynthesis (∼2.5 kb), and an ∼6-kb region encoding pilin and the *haf* pilus export machinery.

**TABLE 1:** Summary of information for the closed annotated genome sequences for five strains of Haemophilus influenzae biogroup *aegyptius*

Strain	Site of isolation	Disease	Genome size (bp)	Genome coverage (×)	Total no. of reads	Avg read length (bp)	GC content (%)	No. of genes	No. of CDS^*a*^	GenBank accession no.	SRA accession no.
F1946	Skin	BPF	1,985,844	128.4	43,712	5,833	38.2	2,028	1,947	CP043770	SRX6897973
F3028	CSF^*b*^	BPF	1,984,979	245.6	101,675	4,796	38.2	2,022	1,941	CP043771	SRX6897974
F3037	Blood	BPF	1,987,687	161.9	56,929	5,655	38.2	2,027	1,946	CP043772	SRX6897975
F3052	Conjunctiva	Conjunctivitis	1,877,864	138.2	34,648	6,488	40.7	1,898	1,822	CP043810	SRX6897976
F3043	Conjunctiva	Conjunctivitis	2,000,194	118.3	39,986	6,829	35.7	2,038	1,958	CP043811	SRX6897977

a CDS, coding DNA sequences.

b CSF, cerebrospinal fluid.

These five closed and annotated genome sequences have more than doubled the number of whole closed annotated Haemophilus influenzae biogroup *aegyptius* genomes that are available in the public domain and will facilitate the elucidation of the exact factors responsible for the unusual virulence of this lineage of Haemophilus influenzae.

### Data availability.

The genomes have been deposited in GenBank. The accession numbers for the closed genomes and raw data (SRA) are provided in Table 1.
